# Examination of Novel Immunomodulatory Effects of L-Sulforaphane

**DOI:** 10.3390/nu13020602

**Published:** 2021-02-12

**Authors:** Nadia Mazarakis, Jeremy Anderson, Zheng Quan Toh, Rachel A. Higgins, Lien Anh Ha Do, Rodney B. Luwor, Kenneth J. Snibson, Tom C. Karagiannis, Paul V. Licciardi

**Affiliations:** 1 Faculty of Veterinary and Agricultural Sciences, University of Melbourne, Parkville, VIC 3010, Australia; nadia.mazarakis@mcri.edu.au (N.M.); ksnibson@unimelb.edu.au (K.J.S.); 2 New Vaccines, Infection and Immunity, Murdoch Children’s Research Institute, Melbourne, VIC 3052, Australia; jeremy.anderson@mcri.edu.au (J.A.); zheng.quantoh@mcri.edu.au (Z.Q.T.); rachel.higgins@mcri.edu.au (R.A.H.); lienanhha.do@mcri.edu.au (L.A.H.D.); 3 Department of Paediatrics, University of Melbourne, Parkville, VIC 3052, Australia; 4 Department of Surgery, Royal Melbourne Hospital, University of Melbourne, Parkville, VIC 3050, Australia; rluwor@unimelb.edu.au; 5 Epigenomic Medicine Program, Alfred Centre, Department of Diabetes, Central Clinical School, Monash University, Melbourne, VIC 3004, Australia; tom.karagiannis@monash.edu

**Keywords:** anti-inflammatory effects, cruciferous vegetables, dendritic cells, immune cells, immunomodulatory effects, L-sulforaphane, monocytes

## Abstract

The dietary isothiocyanate L-sulforaphane (LSF), derived from cruciferous vegetables, is reported to have several beneficial biological properties, including anti-inflammatory and immunomodulatory effects. However, there is limited data on how LSF modulates these effects in human immune cells. The present study was designed to investigate the immunomodulatory effects of LSF (10 µM and 50 µM) on peripheral blood mononuclear cell (PBMC) populations and cytokine secretion in healthy adult volunteers (*n* = 14), in the presence or absence of bacterial (lipopolysaccharide) and viral (imiquimod) toll-like receptor (TLRs) stimulations. Here, we found that LSF reduced pro-inflammatory cytokines interleukin (IL)-6, IL-1β, and chemokines monocyte chemoattractant protein (MCP)-1 irrespective of TLR stimulations. This result was associated with LSF significantly reducing the proportion of natural killer (NK) cells and monocytes while increasing the proportions of dendritic cells (DCs), T cells and B cells. We found a novel effect of LSF in relation to reducing cluster of differentiation (CD) 14^+^ monocytes while simultaneously increasing monocyte-derived DCs (moDCs: lineage-Human Leukocyte Antigen-DR isotype (HLA-DR)^+^CD11b^low-high^ CD11c^high^). LSF was also shown to induce a 3.9-fold increase in the antioxidant response element (ARE) activity in a human monocyte cell line (THP-1). Our results provide important insights into the immunomodulatory effects of LSF, showing in human PBMCs an ability to drive differentiation of monocytes towards an immature monocyte-derived dendritic cell phenotype with potentially important biological functions. These findings provide insights into the potential role of LSF as a novel immunomodulatory drug candidate and supports the need for further preclinical and phase I clinical studies.

## 1. Introduction

Diet plays a major role in maintaining our immune health, with substantial research demonstrating the health benefits of a fiber-rich diet in reducing the burden of inflammatory-driven diseases. L-sulforaphane (LSF) is a compound found in cruciferous vegetables with several beneficial biological properties including antioxidant, chromatin-modifying, anti-microbial, and anti-inflammatory effects [1,2,3,4]. The metabolites of LSF; LSF-glutathione (LSF-GSH), LSF-cysteine (LSF-cys) and LSF-N-Acetyl-L-cysteine (LSF-NAC), have also been suggested to exhibit the same biological effects as LSF [5]. Previous studies have demonstrated important biological effects of LSF and its metabolites however there is limited evidence for the immunomodulatory effects in humans.

It is suggested that LSF modulates the immune response by targeting antigen-presenting cells (APCs), such as monocytes, macrophages, and dendritic cells (DCs). Several lines of evidence support this. In vitro studies have shown that LSF induces the differentiation of human monocyte-derived macrophages from a pro-inflammatory M1 phenotype to an anti-inflammatory M2 phenotype [6]. In contrast, LSF treatment of porcine monocyte-derived DCs (moDCs) inhibited differentiation into mature moDCs when stimulated with lipopolysaccharide (LPS), yet increased their phagocytic capacity [7]. In mice, intraperitoneal administration of LSF (500 µg/day) over 5 days increased total white blood cell counts and the phagocytic activity of peritoneal macrophages as well as reducing tumour necrosis factor (TNF)-α levels induced by LPS stimulation [8]. LSF was found to have direct effects on tolerogenic DCs in colitis-induced mouse models through activation of adenosine monophosphate-activated protein kinase (AMPK) [9] and was shown to protect against prostate cancer in mice through increased interleukin (IL)-12 secretion by DCs, promoting the infiltration of CD57^+^ cytotoxic natural killer (NK) cells and CD3^+^ T cells [10]. In other studies, impaired alveolar macrophage function from patients with chronic obstructive pulmonary disease was restored by LSF treatment as shown by increased bacterial phagocytosis [11]. These studies demonstrate that LSF may exhibit promising immunomodulatory effects through targeting APCs. However, there is limited data in human immune cells for the effects of LSF.

In this study, we investigated the in vitro immunomodulatory effects of LSF on peripheral blood mononuclear cells (PBMCs) from healthy adults using a combination of flow cytometric and cytokine assays.

## 2. Materials and Methods

### 2.1. Reagents

The human acute monocytic leukemia cell line (THP-1) cell line was kindly provided by Dr Adrian Achuthan (Centre for Medical Research, Royal Melbourne Hospital, Melbourne, Victoria, VIC, Australia). LSF was acquired from Sigma-Aldrich (St. Louis, MO, USA) and its metabolites: LSF-GSH, LSF-cys and LSF-NAc were obtained from Santa Cruz (Dallas, TX, USA). Lipopolysaccharide (LPS) and imiquimod (IMQ) were acquired from Jomar Life Research, Scoresby, VIC, Australia. All other study reagents were acquired from Sigma-Aldrich unless otherwise specified.

### 2.2. Study Samples

Heparinized blood samples (20 mL) were collected from 14 healthy adult volunteers (18–50 years old) following written informed consent. This study was approved by the Royal Children’s Hospital Human Research Ethics Committee (HREC# 36236A).

### 2.3. PBMC Culture

PBMCs were isolated from whole blood samples by density gradient centrifugation using Lymphoprep (Alere Technologies AS, Oslo, Norway). 1 × 10^6^ PBMCs/mL in R10 media; RPMI-1640 medium supplemented with 10% fetal bovine serum (Thermo Fisher Scientific, Waltham, MA, USA), 200 nM L-glutamine, 1000 IU penicillin-streptomycin, were pre-treated with 10 μM and 50 μM LSF or metabolites (LSF-cys, LSF-GSH, LSF-NAc) for 24 h followed by stimulation with LPS (10 ng/mL) or IMQ (5 mg/mL) for a further 24 h (*n* = 14) or were left unstimulated (media only). In separate studies, 1 × 10^6^ PBMCs/mL were treated with 10 μM and 50 μM LSF for 6 h, 24 h and 48 h without TLR stimulation (*n* = 8). A control group with R10 media only (for TLR stimulations) and a dimethyl sulfoxide (DMSO)-only group was used (for LSF) in each experiment. Cell viability was measured at these time points using trypan blue exclusion dye. All cultured cells were maintained at 37 °C, 5% CO_2_. Supernatants were collected and stored at −20 °C until cytokine/chemokine measurements. PBMCs were harvested and stained immediately for flow cytometry experiments.

### 2.4. Flow Cytometry

PBMCs were stained with the Zombie Aqua Flexibility Viability dye (BioLegend, San Diego, CA, USA) for the live/dead gate for 15 min at room temperature, followed by the antibodies for (1) TLR stimulation experiments: CD3-PE-Cy7, CD19-APC-H7, CD56-BV421, CD16-BUV395, HLA-DR-BB515, CD11b-BV605, CD11c-APC (all from Becton Dickinson, Franklin Lakes, NJ, USA) and CD14-PEDazzle594 (BioLegend); and (2) time-course experiments: a lineage-negative (lin^−^) cocktail (CD3/CD19/CD20/CD56/CD14/CD16-AF700, Bio-Rad, Hercules, CA, USA), CD14-PEDazzle594, HLA-DR^+^-BB515 (Biolegend), CD123-PE (BioLegend), CD11b-BV605 (Becton Dickinson), CD11c-APC (Becton Dickinson), CD141-BV711 (Becton Dickinson), CD1c-BV421 (BioLegend) and CD1aPerCP/Cy5.5 (Biolegend) for 20 min on ice, washed twice and resuspended in flow cytometry buffer (phosphate-buffered saline (PBS) + 2% fetal bovine serum (FBS)).

All samples were run on an LSR Fortessa X-20 (Becton Dickinson) with a minimum of 100,000 events recorded per sample. All data were analyzed with FlowJo Software v10.7.1 (Ashland, OR, USA).

### 2.5. Chemokine and Cytokine Measurements

IL-6 and TNF-α were measured using a commercial enzyme-linked immunosorbent assay (ELISA) kit following the manufacturer’s instructions (R&D Systems, Minneapolis, MN, USA). Results were measured at an optical density of 450 nm (reference wavelength 630 nm), and concentrations in pg/mL were derived from the standard curve. IL-1β, IL-10, Regulated upon Activation, Normal T Cell Expressed and Presumably Secreted (RANTES) and MCP-1 were measured using a multiplex bead array kit according to the manufacturer’s instructions (Bio-Rad) with results analyzed on a Bio-Plex 200 system instrument (Bio-Rad) fitted with the Bio-Plex Manager v6 software (Bio-Rad) and results reported in pg/mL.

### 2.6. THP-1 Cell Culture

THP-1 cells were maintained with RPMI-1640 media supplemented with 10% Fetal Bovine Serum, 10 mM HEPEs, 1% Penicillin-streptomycin, at 37 °C, 5% CO_2_. THP-1 cells (1 × 10^5^ cells/mL) were treated with 10 µM and 50 µM LSF for 6 h, 24 h and 48 h (*n* = 3). A control group with R10 media only and a DMSO-only group was used as the vehicle control group for each experiment. Viability of THP-1 monocytes was measured using trypan blue exclusion dye at each timepoint.

### 2.7. Antioxidant Response Element (ARE)-Luciferase Reporter Assay

THP-1 cells (1 × 10^5^ cells/mL) were transfected with ARE construct (pGL4.37; Promega, Madison, WI, USA) using FuGENE HD transfection reagent (Promega) following the manufacturer’s instructions. Transfected cells were then seeded into a 96-well flat-bottom plate and incubated for 24 h at 37 °C, 5% CO_2_. After 24 h, 10 µM and 50 µM LSF was added to the transfected cells and incubated for a further 6 h and 24 h. Cells were lysed in lysis buffer (Promega) and assessed for ARE-luciferase activity with the use of the Luciferase Reporter Assay Kit (Promega) and a GloMax 96 Microplate Luminometer (Promega) following the manufacturer’s instructions.

### 2.8. Statistical Analysis

Cytokine/chemokine measurements, flow cytometric results, and cell viability of PBMCs were analyzed using a non-parametric Wilcoxon signed-rank test, and these results were shown as median ± interquartile range (IQR). This was used to avoid any influence of outlier results that would be apparent by using statistical tests based on the mean. THP-1 monocyte ARE-luciferase assay and cell viability assays were analyzed using a Student T-test, with results shown as mean ± 95% confidence interval (CI). All statistical analyses were performed using Prism 8 (GraphPad, San Diego, CA, USA). A *p*-value of less than 0.05 was considered significant in all cases.

## 3. Results

### 3.1. LSF and its Metabolites Reduced Chemokines and Cytokines

**Figure 1:**
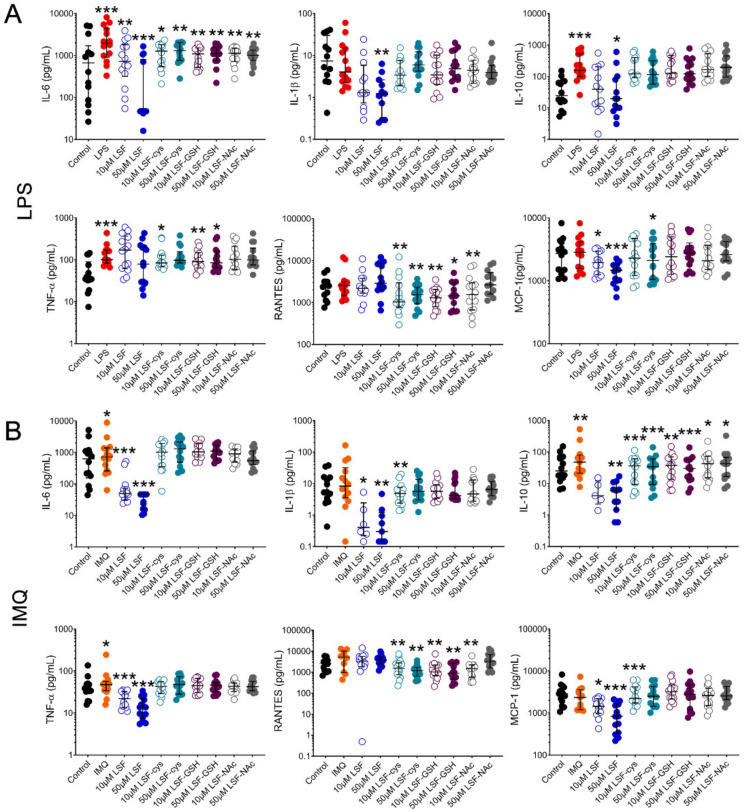
LSF effect on cytokine and chemokine secretion. Cytokine and chemokine measurements in supernatants from healthy adult PBMCs (*n* = 14) following pre-treatment with 10 µM or 50 µM LSF or its metabolites (LSF-cys, LSF-GSH, LSF-NAc) for 24 h before stimulation with LPS (10 ng/mL) (**A**) or IMQ (5 mg/mL) (**B**) for a further 24 h. The medium control was used as the control group for comparisons with the TLR-stimulated groups; the DMSO control was used as the vehicle control for comparison with the LSF treatments. * *p* < 0.05, ** *p* < 0.01, *** *p* < 0.001 compared to the respective stimulation controls, assessed by a non-parametric Wilcoxon signed-rank test. The median ± IQR range is displayed. Legend: • Control, • LPS, • IMQ, • LSF, • LSF-cys, • LSF-GSH, • LSF-NAc (10 µM are open circles and 50 µM are closed circles).Abbreviation: IL, interleukin; IMQ, imiquimod; IQR, inter-quartile range; LPS, lipopolysaccharide; LSF, L-sulforaphane; LSF-cys, LSF-cysteine; LSF-GSH, LSF-glutathione; LSF-NAc, LSF-N-Acetyl-L-cysteine; MCP-1, monocyte chemoattractant protein -1; PBMC, peripheral blood mononuclear cells; RANTES, Regulated upon Activation, Normal T Cell Expressed and Presumably Secreted; TNF-α, tumour necrosis factor-alpha.

We first examined whether pre-treatment of PBMCs with LSF or its metabolites (LSF-cys, LSF-GSH and LSF-NAc) reduced chemokine and cytokine production when stimulated with a bacterial (LPS) or viral (IMQ) ligand (Figure 1). Following stimulation with LPS (Figure 1A), 10 µM and 50 µM LSF significantly reduced IL-6 (*p* < 0.01 and *p* < 0.001, respectively) and MCP-1 (*p* < 0.05 and *p* < 0.001, respectively). In addition, 50 µM LSF also significantly reduced IL-1β (*p* < 0.01) and IL-10 (*p* < 0.05). For IMQ (Figure 1B), 10 µM and 50 µM LSF elicited a dose-related reduction on IL-6 (*p* < 0.001 for each dose), IL-1β (*p* < 0.05 and *p* < 0.01, respectively), IL-10 (not significant (ns) and *p* < 0.01 respectively), TNF-α (*p* < 0.001 for each dose) and MCP-1 (*p* < 0.05 and *p* < 0.01, respectively), but no effect on RANTES. LSF metabolites (LSF-cys, LSF-GSH and LSF-NAc) at both doses significantly reduced IL-6 (*p* < 0.01) after LPS stimulation and IL-10 (*p* < 0.01 for LSF-cys and LSF-GSH, and *p* < 0.05 for LSF-NAc) after IMQ stimulation. LSF-cys and LSF-GSH also reduced TNF-α (*p* < 0.05) after LPS stimulation and RANTES (*p* < 0.01) following LPS or IMQ stimulation, while only the 10 µM dose of LSF-NAc reduced RANTES (*p* < 0.01).

Given the more robust effect for LSF on cytokine and chemokine levels compared with the metabolites, subsequent experiments were focused on LSF only.

### 3.2. LSF Effects on Immune Cell Phenotype

**Figure 2:**
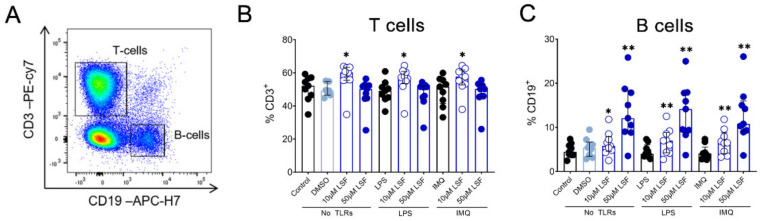
The effect of LSF on adaptive immune cell populations. Flow cytometric gating strategy (**A**) and the proportion of T cells (**B**) and B cells (**C**) (*n* = 9) displayed as a proportion of total lymphocyte populations. The median ± IQR range is presented. The medium control was used as the control group for comparisons with the TLR-stimulated groups; the DMSO control was used as the vehicle control for comparison with the LSF treatments. * *p* < 0.05, ** *p* < 0.01 compared to the respective untreated control, with statistical analysis performed using a non-parametric Wilcoxon signed-rank test. Legend: • Control/LPS/IMQ, 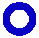 10 µM LSF, • 50 µM LSF, • DMSO. Abbreviation: CD, cluster of differentiation; DMSO, dimethyl sulfoxide; IMQ, imiquimod; IQR, inter-quartile range; LPS, lipopolysaccharide; LSF, L-sulforaphane; TLR, toll-like receptor.

**Figure 3:**
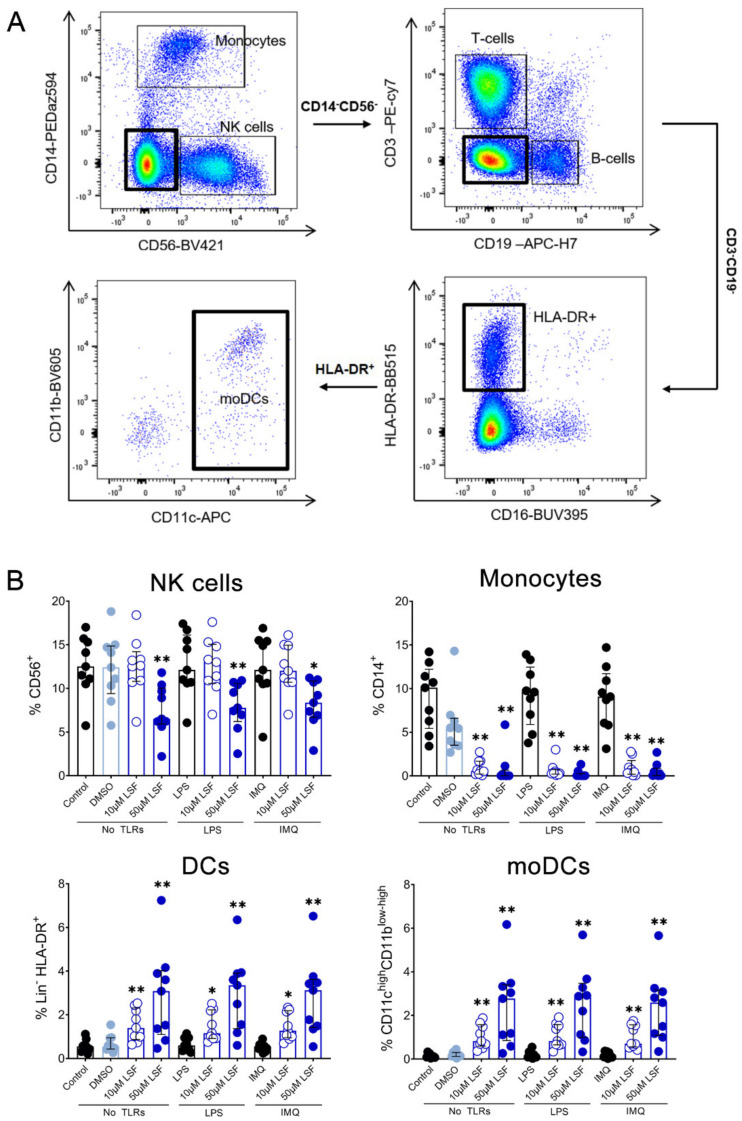
The effect of LSF on innate immune cell populations. Gating strategy (**A**) and flow cytometric results (*n* = 9) are displayed as a proportion of total lymphocyte populations (**B**). The median ± IQR is displayed. The medium control was used as the control group for comparisons with the TLR-stimulated groups; the DMSO control was used as the vehicle control for comparison with the LSF treatments. * *p* < 0.05, ** *p* < 0.01 compared to the respective untreated control using a non-parametric Wilcoxon signed-rank test. Legend: • Control/LPS/IMQ, 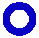 10 µM LSF, • 50 µM LSF, • DMSO. Abbreviation: CD, cluster of differentiation; DC, dendritic cell, DMSO, dimethyl sulfoxide; HLA-DR, Human Leukocyte Antigen–DR isotype; IMQ, imiquimod; IQR, inter-quartile range; Lin, lineage; LPS, lipopolysaccharide; LSF, L-sulforaphane; moDC, monocyte-derived dendritic cell; NK, natural killer cell; TLR, toll-like receptor.

Next, we examined whether the cytokine responses observed were associated with changes in immune cell populations using flow cytometry. Following 10 µM LSF treatment, there was a higher proportion of CD3^+^ T cells (*p* < 0.05) (Figure 2B) and a dose-related increase in CD19^+^ B cells (*p* < 0.01) (Figure 2C) compared to the untreated control cells. At the high dose of 50 µM, LSF significantly reduced CD56^+^ NK cells (*p* < 0.01) as well as reduce CD14^+^ monocytes (*p* < 0.01) with increased dose of LSF as compared to the control groups (Figure 3B). In contrast to the reduced CD14+ monocyte populations, LSF increased the proportion of HLA-DR^+^ DCs (*p* < 0.01) as well as immature moDCs (HLA-DR^+^CD11c^high^ CD11b^low-high^) by 48 h (*p* < 0.01). These effects for LSF appeared to be independent of TLR stimulation since they were also observed in unstimulated cells.

### 3.3. Immunomodulatory Effects of LSF on DC Populations

**Figure 4:**
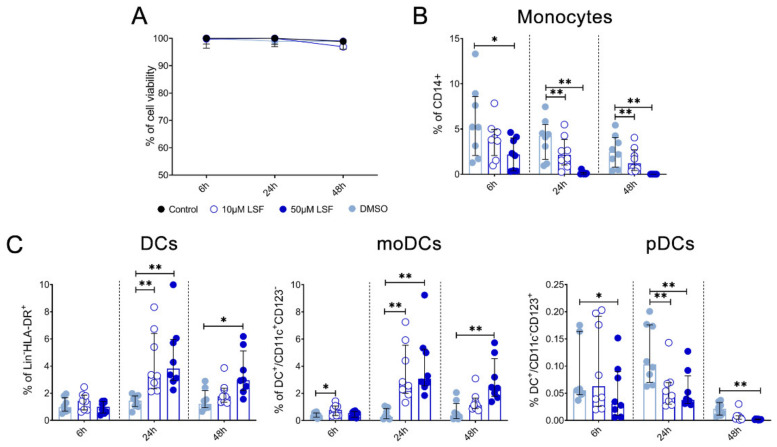
LSF effect on monocyte and dendritic cell populations. The effect of 10 µM or 50 µM LSF on DC populations in healthy adult PBMCs (*n* = 8) over 6, 24, or 48 h. PBMC viability (*n* = 8) (**A**) and flow cytometric results of monocytes (**B**) and DC subsets (**C**) displayed as a proportion of total lymphocyte populations, with the median ± IQR displayed. DMSO was used as a vehicle control for LSF treatments. * *p* < 0.05, ** *p* < 0.01 compared to the untreated DMSO control using a non-parametric Wilcoxon signed-rank test. Legend: • Control, 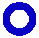 10 µM LSF, • 50 µM LSF, • DMSO. Abbreviation: CD, cluster of differentiation; DC, dendritic cell, DMSO, dimethyl sulfoxide; HLA-DR, Human Leukocyte Antigen – DR isotype; IQR, inter-quartile range; Lin, lineage; LPS, lipopolysaccharide; LSF, L-sulforaphane; moDC, monocyte-derived dendritic cell; PBMC, peripheral blood mononuclear cells; pDC, plasmacytoid dendritic cells.

Given these effects of LSF on broad DC populations, we next performed detailed immune phenotyping of specific DC populations induced by LSF over time (6 h, 24 h and 48 h) (Figure 4). Here, we used the markers Lin^-^HLA-DR^+^CD11c^high^ CD123^−^ to identify moDCs. However, additional markers such as CD1a, CD1c and CD141 did not further discriminate moDC populations (data not shown). LSF treatment did not affect cell viability, with PBMCs >90% viable after 48 h (Figure 4A). Consistent with our earlier observations, LSF significantly reduced CD14^+^ monocytes at the 50 µM dose after 6 h (*p* < 0.05), and both doses at 24 h and 48 h (both *p* < 0.01) (Figure 4B). This was associated with a significant increase in total DCs (Lin-HLA-DR+) at 24 h (*p* < 0.01) and 48 h (*p* < 0.05) compared to the control group (Figure 4C). Consistent with our earlier observations, LSF induced a significant dose-related increase in immature moDCs (Lin-HLA-DR^+^CD11c^high^ CD123^−^) at all timepoints (Figure 4C). In contrast, 50 µM LSF significantly reduced plasmacytoid DC (pDC) numbers at all timepoints (*p* < 0.05) (Figure 4C).

**Figure 5:**
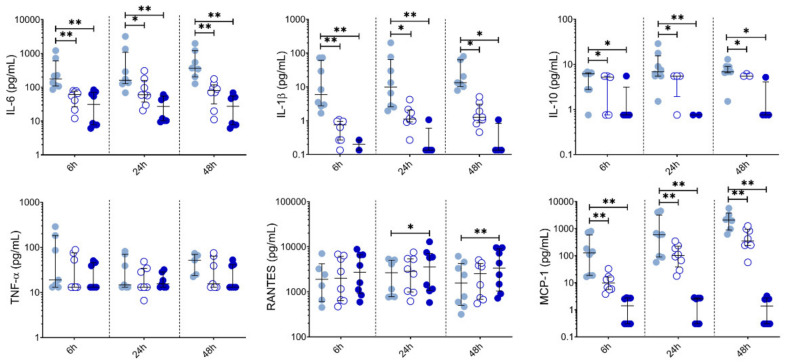
LSF effect on cytokine and chemokine secretion over time. Cytokine and chemokine measurements in supernatants from healthy adult PBMCs (*n* = 8) following treatment with 10 µM or 50 µM LSF for 6 h, 24h, or 48 h. The median ± IQR are displayed. DMSO was used as a vehicle control for LSF treatments. * *p* < 0.05, ** *p* < 0.01 compared to the untreated DMSO control using a non-parametric Wilcoxon signed-rank test. Legend: • Control, 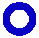 10 µM LSF, • 50 µM LSF, • DMSO. Abbreviation: DMSO, dimethyl sulfoxide; IL, interleukin; IQR, inter-quartile range; LSF, L-sulforaphane; MCP-1, monocyte chemoattractant protein-1; RANTES, PBMC, peripheral blood mononuclear cells; Regulated upon Activation, Normal T Cell Expressed and Presumably Secreted; TNF-α, tumour necrosis factor-alpha.

We also measured chemokine and cytokine levels in PBMC supernatants collected at 6 h, 24 h and 48 h (Figure 5). Both 10 µM and 50 µM LSF significantly reduced IL-6 (*p* < 0.05), IL-1β (*p* < 0.05), IL-10 (*p* < 0.05) and MCP-1 (*p* < 0.01) across all timepoints. RANTES was significantly increased at 24 h and 48 h by 50 µM LSF (*p* < 0.05, and *p* < 0.01), while 10 µM LSF significantly increased in RANTES only after 48 h (*p* < 0.05). No effect of LSF was observed for TNF-α.

**Figure 6:**
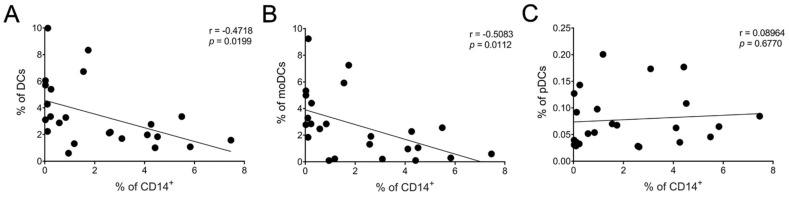
Association between reduced CD14+ cells and increased DC populations. Correlation between CD14^+^ cells and DC subsets: (**A**) total DCs, (**B**) moDCs, and (**C**) pDCs after 24 h of LSF (10 µM and 50 µM) treatment. Each datapoint is an individual sample across each of the three groups (*n* = 8/group). A Pearson’s correlation was performed. Abbreviation: CD, cluster of differentiation; DC, dendritic cell, LSF, L-sulforaphane; moDC, monocyte-derived dendritic cell; pDC, plasmacytoid dendritic cells.

There was a moderate negative but statistically significant (*p* < 0.05) correlation between the frequency of CD14^+^ cells and total DCs (Figure 6A) as well as immature moDCs (Figure 6B). No statistical significance was identified between CD14^+^ and pDCs (Figure 6C). We also found a moderate correlation between the frequency of CD14^+^ cells and RANTES level (* *p* < 0.05) (Supplementary Figure S1) but not for any of the other cytokines/chemokines measured.

### 3.4. LSF Increases Nrf2-ARE Activity in THP-1 Monocytes

**Figure 7:**
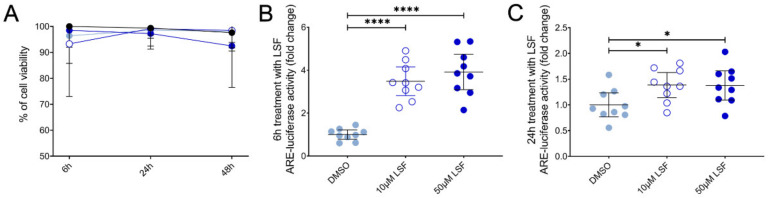
The effect of 10 µM or 50 µM LSF on Nrf2-ARE activity in THP-1 monocytes. Cell viability (**A**) and ARE-luciferase reporter activity after 6 h (**B**) and 24 h (**C**) of LSF treatment are shown. Data was collected as three independent experiments. The mean ± 95% CI are displayed. DMSO was used a vehicle control for LSF treatments. * *p* < 0.05, **** p < 0.0001 compared to the untreated DMSO control cells using the Student’s *t*-test. Legend: • Control, 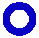 10 µM LSF, • 50 µM LSF, • DMSO. Abbreviation: ARE, antioxidant response element; CI, confidence interval; DMSO; dimethyl sulfoxide; LSF, L-sulforaphane.

We next examined Nrf2-ARE activity in THP-1 monocytes as the basis for these effects and found that LSF significantly increased Nrf2-ARE activity by 3.9-fold (*p* < 0.001) after 6 h (Figure 7B), and 1.4-fold after 24 h (Figure 7C) (*p* < 0.01) compared to the untreated control group.

## 4. Discussion

Understanding the health-promoting effects of dietary compounds has broad public and scientific interest. LSF has gained immense interest for its multifactorial beneficial effects, particularly the significant clinical utility associated with its anti-inflammatory effects. Despite this, little is known about the effects of LSF on human immune cell populations. In this study, we showed that LSF has potent immunomodulatory effects, and report two key findings: 1) that LSF reduces pro-inflammatory cytokine and chemokine production in TLR-stimulated PBMCs and 2) we describe for the first time in healthy human PBMCs that LSF mediates its effects primarily on monocytes and drives their differentiation into an immature moDC population irrespective of TLR stimulation. Our results have important implications for the potential use of LSF in a range of clinical applications.

LSF is reported to have anti-inflammatory effects through the inhibition of nuclear factor kappa Beta (NF-κΒ), [7,12,13,14]. In this study, LSF significantly decreased IL-6, IL-10, IL-1β and MCP-1 following both viral (IMQ) and bacterial (LPS) stimulations, consistent with previous in vitro and in vivo studies [7,15,16,17]. The doses of LSF used in this study were consistent with several previously published reports [7,15,18,19,20] despite being higher than levels detected in blood [21,22,23,24]. Determining the precise biologically relevant concentrations of LSF needed to exert clinical effects in vivo is a critical next step for this research. However, less is known about these effects for the metabolites of LSF. We found that LSF metabolites exhibited anti-inflammatory effects by reducing IL-6, TNF-α, IL-10, and RANTES after TLR stimulations but overall, this was less robust than what we observed for LSF.

Interestingly, RANTES was the only chemokine that LSF did not affect following TLR stimulation, yet LSF increased RANTES in the absence of TLR stimulation. RANTES is a chemokine that is usually expressed in the late phase immune response following T-cell activation [25] and not activated through the conventional Janus kinase (JAK)/signal transducer and activator of transcription (STAT) (JAK/STAT) pathways involving NF-κΒ [26,27]. The fact that the metabolites but not LSF were able to reduce RANTES points to a possible alternative mechanism to explain this. For example, LSF metabolites may be less effective at targeting Nrf2 or inhibiting NF-kB, although, more detailed investigations are needed to confirm this result as well as their potential clinical relevance.

A key observation from our study was that LSF reduced the frequency of CD14^+^ monocytes while simultaneously increasing immature moDCs in the presence and absence of TLR stimulations. This result was supported by a moderate negative correlation found between the frequency of CD14^+^ cells and total DCs as well as immature moDCs after 24h of LSF treatment. One caveat to these findings is that these immature moDCs were characterized as Lin^-^CD14^-^HLA-DR^+^CD11c^high^ CD11b^low-high^, which in the blood are indistinguishable from myeloid DCs (mDCs) with this phenotype [27]. Additional markers such as CD1c (mDC1), CD141 (mDC2) and CD1a (moDC) to identify moDCs further were not informative. However, we believe that these are likely to be moDCs rather than mDCs since this immature moDC differentiation from monocytes has been reported elsewhere [28,29]. A similar result in CD14^+^ enriched cells was reported by Kumar et al. [20], where they concluded that the increased CD14^-^HLA-DR^+^ frequency after LSF treatment was considered an immature moDC population as they lacked the expression of maturation markers CD80 and CD83. Although we did not look specifically at the expression of maturation markers, it is plausible that the increase in DCs population we observed are immature moDCs [27,28,29].

To further investigate this LSF effect on monocytes specifically, we measured Nrf2-ARE activity using a luciferase reporter assay. Nrf2-ARE activation is a hallmark characteristic of LSF function [2,5,30,31,32] and is also a crucial component of cell differentiation in various cell types, such as human hematopoietic stem cells, promyelocytes (HL-60) and monocytes (U937) [33,34,35]. Our data provides support to the idea that LSF induces immunomodulatory effects through activation of Nrf2-ARE, leading to changes in monocyte phenotype and function towards an immature moDC pathway.

MoDCs are classified as immunogenic (mature) or tolerogenic (immature) [36]. During infection, moDCs are recruited to the site of inflammation and matured upon TLR stimulation to prompt a pro-inflammatory cytokine/chemokine response, with the secretion of IL-6, MCP-1, and TNF-α [37,38]. Our data, however, displayed a down-regulation of these pro-inflammatory cytokines/chemokines irrespective of TLR stimulations. Therefore, based on our data, we hypothesize that LSF may drive the development of a tolerogenic immature moDC phenotype that possesses immunomodulatory activity [27,38]. This response is not surprising considering that LSF inhibits NF-κΒ, and NF-κΒ activation is required for DC maturation to an immunogenic phenotype [39,40]. Although tolerogenic moDCs typically produce IL-10, we did not observe this in our study. There is, however, growing evidence for the diversity of in vitro formed tolerogenic moDCs phenotypes, including specific populations that do not secret IL-10 [41].

Immature moDCs are also potent phagocytes, can cross-present antigens, inhibit T cell proliferation and increase regulatory cells which are crucial for immune tolerance and protection against autoimmune diseases [36,42,43,44,45]. In a study by Qu et al., LSF was shown to inhibit the maturation of immature porcine-derived moDC while increasing their phagocytic capacity [7]. In humans, LSF reversed impaired alveolar macrophage function in chronic obstructive pulmonary disease (COPD) patients by increasing the phagocytosis of non-typeable *Haemophilus influenzae* through the up-regulation of the Nrf2-ARE pathway [11]. Further investigation of the phagocytic effect of the LSF-induced immature moDCs would therefore be of great interest.

We also observed significant phenotypic changes following LSF treatment and TLR stimulations this included a rise in T and B cells. These changes might be due to a rise in tolerogenic moDCs which can promote an increase in T and B regulatory cells, although further studies are needed to confirm this [36,42,43]. Furthermore, NK cells and pDC populations were also significantly decreased following LSF treatment. More intriguing, however, is that pDC population are a significant source of the anti-viral cytokine IFNα/β and NK cells are also mediate anti-viral responses [46,47]. Therefore, our data suggests that LSF may have an impact on reducing anti-viral activity [48] mediated through the increase of moDCs, although this needs to assessed in relevant in vivo models.

Tolerogenic moDCs-therapies have become an attractive area of research for allergic diseases, autoimmune diseases, cancer as well as protecting against organ transplantation rejection [36,45,49]. Thus far, early Phase I/II trials have yielded promising results [50,51]. Current protocols for developing tolerogenic moDCs include stimulating isolated monocytes with IL-4 and granulocyte-macrophage colony-stimulating factor (GM-CSF) [28], vitamin D3, dexamethasone [41], or IL-10 [52,53]. LSF offers a potential promising alternative for developing tolerogenic moDC-therapies. Therefore, further functional assessment of LSF-induced moDCs would add valuable insight into their potential therapeutic impact.

This study has several limitations. Although our study had a relatively small sample size, the effect size observed for LSF was consistent and significant in the same direction across different experiments. Although our phenotyping strategy including the use of moDC specific markers CD1c, CD141, and CD1a was unable to conclusively identify these populations, our data suggest that this is likely to be immature moDCs based on their cytokine profiles. We did not assess the phagocytic potential of these immature moDCs as this was beyond the scope of this study but would be an important part of future work. Further studies to examine effects on NF-κΒ pathway or single-cell RNA-seq studies may be more robust approaches to characterize these immature moDC subsets.

## 5. Conclusions

Overall, our findings present novel insights into the effects of the dietary-isothiocyanate LSF on human immune responses. LSF possesses potent immunomodulatory and anti-inflammatory effects on the phenotype and function of moDC populations that suggest it could be an attractive therapeutic candidate for immune-mediated conditions. Moreover, targeting moDCs by LSF might also be a valuable immunotherapy approach but further research in relevant models is needed.

## Supplementary Materials

The following are available online at https://www.mdpi.com/2072-6643/13/2/602/s1, Figure S1: Correlation between CD14+ and cytokine/chemokine production after 24 h of LSF (10 µM and 50 µM) treatment.

## References

[1] Fahey J.W., Talalay P. (1999). Antioxidant functions of sulforaphane: A potent inducer of Phase II detoxication enzymes. Food Chem. Toxicol..

[2] Ho E., Clarke J.D., Dashwood R.H. (2009). Dietary sulforaphane, a histone deacetylase inhibitor for cancer prevention. J. Nutr..

[3] Yanaka A. (2017). Role of Sulforaphane in Protection of Gastrointestinal Tract Against H. pylori and NSAID-Induced Oxidative Stress. Curr. Pharm. Des..

[4] Heiss E., Herhaus C., Klimo K., Bartsch H., Gerhäuser C. (2001). Nuclear factor kappa B is a molecular target for sulforaphane-mediated anti-inflammatory mechanisms. J. Biol. Chem..

[5] Myzak M.C., Karplus P.A., Chung F.L., Dashwood R.H. (2004). A novel mechanism of chemoprotection by sulforaphane: Inhibition of histone deacetylase. Cancer Res..

[6] Pal S., Konkimalla V.B. (2016). Sulforaphane regulates phenotypic and functional switching of both induced and spontaneously differentiating human monocytes. Int. Immunopharmacol..

[7] Qu X., Pröll M., Neuhoff C., Zhang R., Cinar M.U., Hossain M.M., Tesfaye D., Große-Brinkhaus C., Salilew-Wondim D., Tholen E. (2015). Sulforaphane epigenetically regulates innate immune responses of porcine monocyte-derived dendritic cells induced with lipopolysaccharide. PLoS ONE.

[8] Thejass P., Kuttan G. (2007). Immunomodulatory activity of Sulforaphane, a naturally occurring isothiocyanate from broccoli (Brassica oleracea). Phytomedicine.

[9] Deng Z., Rong Y., Teng Y., Mu J., Zhuang X., Tseng M., Samykutty A., Zhang L., Yan J., Miller D. (2017). Broccoli-Derived Nanoparticle Inhibits Mouse Colitis by Activating Dendritic Cell AMP-Activated Protein Kinase. Mol. Ther..

[10] Singh S.V., Warin R., Xiao D., Powolny A.A., Stan S.D., Arlotti J.A., Zeng Y., Hahm E.R., Marynowski S.W., Bommareddy A.D. (2009). Sulforaphane inhibits prostate carcinogenesis and pulmonary metastasis in TRAMP mice in association with increased cytotoxicity of natural killer cells. Cancer Res..

[11] Harvey C.J., Thimmulappa R.K., Sethi S., Kong X., Yarmus L., Brown R.H., Feller-Kopman D., Wise R., Biswal S. (2011). Targeting Nrf2 signaling improves bacterial clearance by alveolar macrophages in patients with COPD and in a mouse model. Sci. Transl. Med..

[12] Neumann M., Naumann M. (2007). Beyond IkappaBs: Alternative regulation of NF-kappaB activity. FASEB J..

[13] Afonina I.S., Zhong Z., Karin M., Beyaert R. (2017). Limiting inflammation-the negative regulation of NF-κB and the NLRP3 inflammasome. Nat. Immunol..

[14] Bessler H., Djaldetti M. (2018). Broccoli and human health: Immunomodulatory effect of sulforaphane in a model of colon cancer. Int. J. Food Sci. Nutr..

[15] Reddy S.A., Shelar S.B., Dang T.M., Lee B.N., Yang H., Ong S.M., Ng H.L., Chui W.K., Wong S.C., Chew E.H. (2015). Sulforaphane and its methylcarbonyl analogs inhibit the LPS-stimulated inflammatory response in human monocytes through modulating cytokine production, suppressing chemotactic migration and phagocytosis in a NF-κB- and MAPK-dependent manner. Int. Immunopharmacol..

[16] Subedi L., Lee J.H., Yumnam S., Ji E., Kim S.Y. (2019). Anti-Inflammatory Effect of Sulforaphane on LPS-Activated Microglia Potentially through JNK/AP-1/NF-κB Inhibition and Nrf2/HO-1 Activation. Cells.

[17] Yang Q., Pröll M.J., Salilew-Wondim D., Zhang R., Tesfaye D., Fan H., Cinar M.U., Große-Brinkhaus C., Tholen E., Islam M.A. (2016). LPS-induced expression of CD14 in the TRIF pathway is epigenetically regulated by sulforaphane in porcine pulmonary alveolar macrophages. Innate Immun..

[18] Vuong L.D., Nguyen Q.N., Truong V.L. (2019). Anti-inflammatory and anti-oxidant effects of combination between sulforaphane and acetaminophen in LPS-stimulated RAW 264.7 macrophage cells. Immunopharmacol. Immunotoxicol..

[19] Bewley M.A., Budd R.C., Ryan E., Cole J., Collini P., Marshall J., Kolsum U., Beech G., Emes R.D., Tcherniaeva I. (2018). Opsonic Phagocytosis in Chronic Obstructive Pulmonary Disease Is Enhanced by Nrf2 Agonists. Am. J. Respir. Crit Care Med..

[20] Kumar R., de Mooij T., Peterson T.E., Kaptzan T., Johnson A.J., Daniels D.J., Parney I.F. (2017). Modulating glioma-mediated myeloid-derived suppressor cell development with sulforaphane. PLoS ONE.

[21] Atwell L.L., Hsu A., Wong C.P., Stevens J.F., Bella D., Yu T.-W., Pereira C.B., Löhr C.V., Christensen J.M., Dashwood R.H. (2015). Absorption and chemopreventive targets of sulforaphane in humans following consumption of broccoli sprouts or a myrosinase-treated broccoli sprout extract. Mol. Nutr. Food Res..

[22] Ye L., Dinkova-Kostova A.T., Wade K.L., Zhang Y., Shapiro T.A., Talalay P. (2002). Quantitative determination of dithiocarbamates in human plasma, serum, erythrocytes and urine: Pharmacokinetics of broccoli sprout isothiocyanates in humans. Clin. Chim. Acta.

[23] Sivapalan T., Melchini A., Saha S., Needs P.W., Traka M.H., Tapp H., Dainty J.R., Mithen R.F. (2018). Bioavailability of Glucoraphanin and Sulforaphane from High-Glucoraphanin Broccoli. Mol. Nutr. Food Res..

[24] Vermeulen M., Klöpping-Ketelaars I.W., van den Berg R., Vaes W.H. (2008). Bioavailability and kinetics of sulforaphane in humans after consumption of cooked versus raw broccoli. J. Agric. Food Chem..

[25] Schall T.J., Jongstra J., Dyer B.J., Jorgensen J., Clayberger C., Davis M.M., Krensky A.M. (1988). A human T cell-specific molecule is a member of a new gene family. J. Immunol..

[26] Krensky A.M., Ahn Y.T. (2007). Mechanisms of disease: Regulation of RANTES (CCL5) in renal disease. Nat. Clin. Pract. Nephrol..

[27] Collin M., Bigley V. (2018). Human dendritic cell subsets: An update. Immunology.

[28] Posch W., Lass-Flörl C., Wilflingseder D. (2016). Generation of Human Monocyte-derived Dendritic Cells from Whole Blood. J. Vis. Exp..

[29] Boyette L.B., Macedo C., Hadi K., Elinoff B.D., Walters J.T., Ramaswami B., Chalasani G., Taboas J.M., Lakkis F.G., Metes D.M. (2017). Phenotype, function, and differentiation potential of human monocyte subsets. PLoS ONE.

[30] Mulcahy R.T., Wartman M.A., Bailey H.H., Gipp J.J. (1997). Constitutive and beta-naphthoflavone-induced expression of the human gamma-glutamylcysteine synthetase heavy subunit gene is regulated by a distal antioxidant response element/TRE sequence. J. Biol. Chem..

[31] Itoh K., Wakabayashi N., Katoh Y., Ishii T., O’Connor T., Yamamoto M. (2003). Keap1 regulates both cytoplasmic-nuclear shuttling and degradation of Nrf2 in response to electrophiles. Genes Cells.

[32] Dinkova-Kostova A.T., Holtzclaw W.D., Cole R.N., Itoh K., Wakabayashi N., Katoh Y., Yamamoto M., Talalay P. (2002). Direct evidence that sulfhydryl groups of Keap1 are the sensors regulating induction of phase 2 enzymes that protect against carcinogens and oxidants. Proc. Natl. Acad. Sci. USA.

[33] Bobilev I., Novik V., Levi I., Shpilberg O., Levy J., Sharoni Y., Studzinski G.P., Danilenko M. (2011). The Nrf2 transcription factor is a positive regulator of myeloid differentiation of acute myeloid leukemia cells. Cancer Biol..

[34] Chen H., Zhang B., Yuan X., Yao Y., Zhao H., Sun X., Zheng Q. (2013). Isoliquiritigenin-induced effects on Nrf2 mediated antioxidant defence in the HL-60 cell monocytic differentiation. Cell Biol. Int..

[35] Murakami S., Shimizu R., Romeo P.H., Yamamoto M., Motohashi H. (2014). Keap1-Nrf2 system regulates cell fate determination of hematopoietic stem cells. Genes Cells.

[36] Marín E., Cuturi M.C., Moreau A. (2018). Tolerogenic Dendritic Cells in Solid Organ Transplantation: Where Do We Stand?. Front. Immunol..

[37] Chow K.V., Sutherland R.M., Zhan Y., Lew A.M. (2017). Heterogeneity, functional specialization and differentiation of monocyte-derived dendritic cells. Immunol. Cell Biol..

[38] Sauter A., Yi D.H., Li Y., Roersma S., Appel S. (2019). The Culture Dish Surface Influences the Phenotype and Cytokine Production of Human Monocyte-Derived Dendritic Cells. Front. Immunol..

[39] Yeang H.X.A., Hamdam J.M., Al-Huseini L.M., Sethu S., Djouhri L., Walsh J., Kitteringham N., Park B.K., Goldring C.E., Sathish J.G. (2012). Loss of transcription factor nuclear factor-erythroid 2 (NF-E2) p45-related factor-2 (Nrf2) leads to dysregulation of immune functions, redox homeostasis, and intracellular signaling in dendritic cells. J. Biol. Chem..

[40] Al-Huseini L.M., Yeang H.X.A., Sethu S., Alhumeed N., Hamdam J.M., Tingle Y., Djouhri L., Kitteringham N., Park B.K., Goldring C.E. (2013). Nuclear factor-erythroid 2 (NF-E2) p45-related factor-2 (Nrf2) modulates dendritic cell immune function through regulation of p38 MAPK-cAMP-responsive element binding protein/activating transcription factor 1 signaling. J. Biol. Chem..

[41] Unger W.W.J., Laban S., Kleijwegt F.S., van der Slik A.R., Roep B.O. (2009). Induction of Treg by monocyte-derived DC modulated by vitamin D3 or dexamethasone: Differential role for PD-L1. Eur. J. Immunol..

[42] Raker V.K., Domogalla M.P., Steinbrink K. (2015). Tolerogenic Dendritic Cells for Regulatory T Cell Induction in Man. Front. Immunol..

[43] Maldonado R.A., von Andrian U.H. (2010). How tolerogenic dendritic cells induce regulatory T cells. Adv. Immunol..

[44] López-Relaño J., Martín-Adrados B., Real-Arévalo I., Lozano-Bartolomé J., Abós B., Sánchez-Ramón S., Alonso B., del Moral M.G., Martínez-Naves E. (2018). Monocyte-Derived Dendritic Cells Differentiated in the Presence of Lenalidomide Display a Semi-Mature Phenotype, Enhanced Phagocytic Capacity, and Th1 Polarization Capability. Front. Immunol..

[45] Domogalla M.P., Rostan P.V., Raker V.K., Steinbrink K. (2017). Tolerance through Education: How Tolerogenic Dendritic Cells Shape Immunity. Front. Immunol..

[46] Van Erp A.E., van Kampen M.R., van Kasteren P.B., de Wit J. (2019). Viral Infection of Human Natural Killer Cells. Viruses.

[47] Hammer Q., Rückert T., Romagnani C. (2018). Natural killer cell specificity for viral infections. Nat. Immunol..

[48] Waisman A., Lukas D., Clausen B.E., Yogev N. (2017). Dendritic cells as gatekeepers of tolerance. Semin. Immunopathol..

[49] Yoo S., Ha S.-J. (2016). Generation of Tolerogenic Dendritic Cells and Their Therapeutic Applications. Immune Netw..

[50] Benham H., Nel H.J., Law S.C., Mehdi A.M., Street S., Ramnoruth N., Pahau H., Lee B.T., Ng J., Brunck M.E. (2015). Citrullinated peptide dendritic cell immunotherapy in HLA risk genotype-positive rheumatoid arthritis patients. Sci. Transl. Med..

[51] Anderson A.E., Swan D.J., Wong O.Y., Buck M., Eltherington O., Harry R.A., Patterson A.M., Pratt A.G., Reynolds G., Doran J.P. (2017). Tolerogenic dendritic cells generated with dexamethasone and vitamin D3 regulate rheumatoid arthritis CD4(+) T cells partly via transforming growth factor-β1. Clin. Exp. Immunol..

[52] Kryczanowsky F., Raker V., Graulich E., Domogalla M.P., Steinbrink K. (2016). IL-10-Modulated Human Dendritic Cells for Clinical Use: Identification of a Stable and Migratory Subset with Improved Tolerogenic Activity. J. Immunol..

[53] Boks M.A., Kager-Groenland J.R., Haasjes M.S., Zwaginga J.J., van Ham S.M., Brinke A.t. (2012). IL-10-generated tolerogenic dendritic cells are optimal for functional regulatory T cell induction--A comparative study of human clinical-applicable DC. Clin. Immunol..

