# Supplementary material for: Examination of Novel Immunomodulatory Effects of L-Sulforaphane
Source: Nutrients. 2021 Feb 12;13(2):602. doi: 10.3390/nu13020602 (PMC7917832; doi:10.3390/nu13020602)
Supplement: Supplementary file 1 [file nutrients-13-00602-s001.pdf]

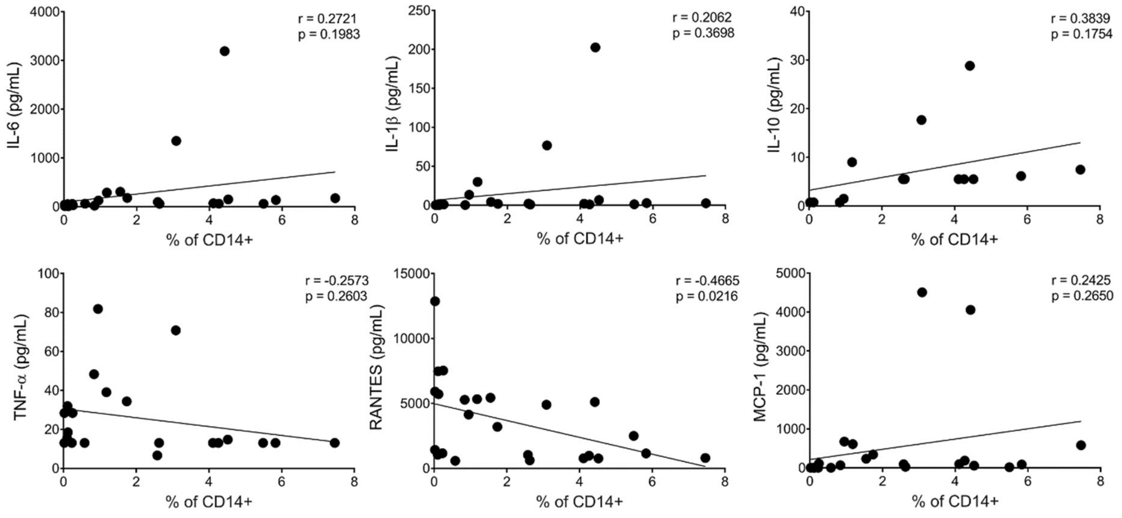

Supplementary Figure S1. Correlation between $CD14^+$ and cytokine/chemokine production after 24 h of LSF (10 μM and 50 μM) treatment. Each datapoint is an individual sample across each of the three groups (*n* = 8/group). A Pearson's correlation was performed. Abbreviation: CD, cluster of differentiation; IL, interleukin; LSF, L-sulforaphane; MCP-1, monocyte chemoattractant protein -1; RANTES, Regulated upon Activation, Normal T Cell Expressed and Presumably Secreted; TNF-α, tumour necrosis factor-alpha.
